# Study on the Consolidation Characteristics of Slurry-like Mud Treated by Flocculation–Solidification–High-Pressure Filtration Combined Method

**DOI:** 10.3390/ma17204992

**Published:** 2024-10-12

**Authors:** Shiliang Li, Yingchao Gao, Rongjun Zhang, Zhekun Zhao

**Affiliations:** 1 China Railway Siyuan Survey and Design Group Co., Ltd., Wuhan 430063, China; 2 School of Civil Engineering, Wuhan University, Wuhan 430072, China

**Keywords:** FSHCM-treated MS, consolidation characteristics, curing agent, initial water content, dry weight of MS

## Abstract

The disposal and reutilization of the enormous amounts of slurry-like mud (MS) dredged from navigation channel construction, ecological dredging, and other construction activities have been receiving increasing attention. In this paper, a flocculation–solidification–high-pressure filtration combined method (FSHCM) is used to treat MS, and the consolidation characteristics of The SHCM-treated MS are studied by conducting a series of one-dimensional consolidation compression tests. Various parameters, including the dosage of the curing agent, initial water content, and dry weight of the MS, are systematically analyzed to evaluate their influence on the consolidation behavior. The experimental results demonstrate that higher curing agent and initial water contents enhance the structural yield stress and compressive resistance, while increased dry weight decreases the structural yield stress but increases the compressive strain and void ratio. As the curing age increases, the ability of the FSHCM-treated MS to resist compressive deformation is further enhanced. In addition, the compressibility of the mud cake samples changes significantly at the yield point. This study has practical guiding significance for the optimal design and long-term application of FSHCM-treated MS.

## 1. Introduction

Enormous amounts of slurry-like mud (MS) are hydraulically dredged annually from navigation channel construction, ecological dredging, and other construction activities [1,2,3,4,5]. The dredged MS is characterized by high water content, high compressibility, poor permeability, and low strength [6,7,8,9]. Traditional disposal methods like inland deposits and ocean dumping are becoming unpopular due to their environmental harm and high costs [10,11,12]. Therefore, it is crucial to develop an efficient and cost-effective method for treating large volumes of MS to enable their utilization as a resource.

Chemical solidification and flocculation conditioning have been widely used in the treatment of MS. Chemical curing technology can be employed to stabilize MS, for example, by using the commonly used curing agent, cement, which is a powdery substance primarily composed of calcined lime and clay. Cement stabilization of MS occurs through the hydration reaction of cement, leading to the formation of calcium silicate hydrate (CSH), calcium aluminate hydrate (CAH), and calcium aluminosilicate hydrate (CASH) [13,14]. It should be noted that the effectiveness of chemical curing technology is significantly influenced by the water content of MS. A higher water content in MS results in a lower curing efficiency [15,16,17,18]. Flocculation conditioning promotes the aggregation of small soil particles into larger flocculent particles by dissolving flocculants in water. This process facilitates rapid precipitation through mechanisms such as network capture and sweeping, adsorption and bridging, and electrical neutralization, ultimately achieving effective dehydration. Flocculation conditioning has problems such as requiring large dosages, being difficult to degrade, and causing environmental pollution [19,20,21]. In addition, pressure filtration dehydration is a commonly used sludge treatment technology [22]. The mechanical properties of MS after pressure filtration remain poor, and it easily reverts to a muddy state when exposed to water [23]. Single mud treatment technologies, such as chemical curing, flocculation conditioning, and pressure filtration, have limitations and struggle to meet complex engineering needs and environmental requirements.

To achieve environmental protection goals, scholars have studied the combination of various treatment technologies that reduce the water content of the slurries and enhance the strength of the treated MS. The flocculation–solidification combined method (FSCM) has been widely used in mud treatment [24]. Through a large number of experiments, Zhang et al. [25] confirmed that the FSCM showed significant advantages over the conventional cement stabilization method (CCSM) in treating high-water-content slurries, with the solidification efficiency of the FSCM at least 4.8 times higher than that of the CCSM. Qin et al. [26] introduced the construction waste-slag-based flocculation–solidification combined method (CWS-FSCM) according to the traditional FSCM, and its feasibility was validated through field tests. The CWS-FSCM demonstrated superior performance in curing effects, carbon emissions, and economic efficiency compared to the traditional FSCM. Sun et al. [27] utilized a geopolymeric flocculation–solidification agent (GBFS-FS) to solidify the geopolymer of tailings mud after sand removal from shield soil. The results indicate that adding GBFS-FS to the mud effectively dehydrates it and produces solidified clay deposits with rapidly increasing strength. Additionally, according to the properties of slurries after flocculation and filtration dehydration, Min et al. [28] found that both inorganic and organic flocculants could improve the pressure filtration dehydration performance of the slurry. Sun et al. [29] investigated the effectiveness of using a gelling agent and geopolymer in conditioning waste slurry through multi-stage pressure filtration, showing that it efficiently dehydrates high-water-content waste slurry and transforms it into high-strength building materials. Han et al. [30] conducted a series of experimental studies to investigate the mechanical properties of MS treated by the flocculation–solidification–high-pressure filtration combined method (FSHCM), and the feasibility of the FSHCM has been demonstrated. However, the consolidation characteristics of MS treated by this combined method are not well understood.

This study focuses on the consolidation characteristics of MS treated by the FSHCM. A series of one-dimensional consolidation compression tests were carried out on FSHCM-treated MS, and the influences of the curing agent contents, initial water contents, and dry weight of the MS on the consolidation characteristics of the FSHCM-treated MS are investigated.

## 2. Laboratory Experiments

### 2.1. Materials

**Figure 1:**
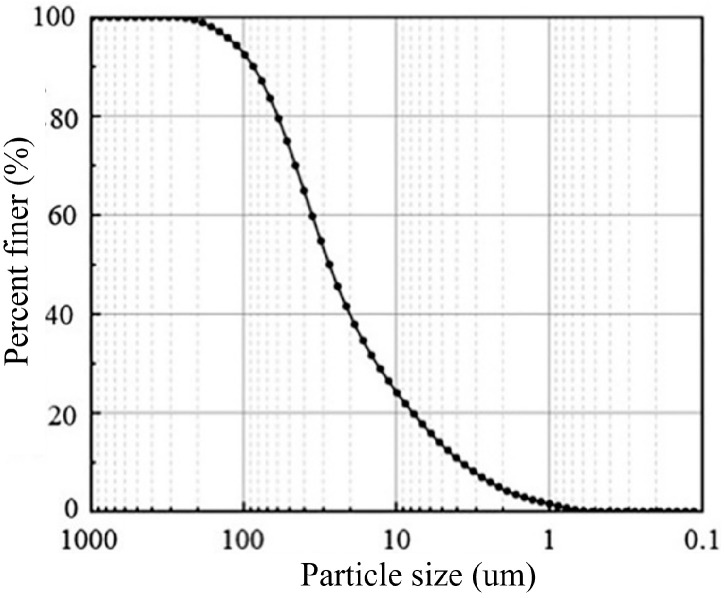
Particle size distribution curve of the tested mud.

**Figure 2:**
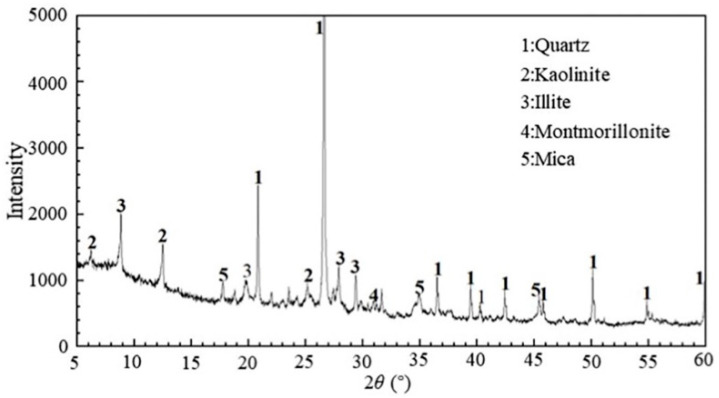
XRD pattern of the tested mud.

The materials used in this study include MS, cement, ground granulated blast-furnace slag (GGBS), flocculant, and water. The MS sample was obtained from a marine deposit in Wenzhou, China. The basic physical properties of the MS are listed in Table 1. The particle size of the mud was measured using a High-Resolution ELPI^®^+ particle size instrument from Dekati Ltd., Kangasala, Finland. The obtained particle size distribution curve is shown in Figure 1. The sand fraction (0.075–2 mm) is 0.8%, the silt fraction (0.002–0.075 mm) is 83.2%, and the clay and colloid fraction (<0.002 mm) is 16.0%. According to the Soil Engineering Classification Standard [31], the mud sample is classified as high liquid limit clay (CH). In addition, the composition of the mud was analyzed through 09Empyrean X-ray diffractometer, and the X-ray diffraction pattern obtained is shown in Figure 2.

The composite curing agents used in this study are P.O.42.5 Ordinary Portland Cement (OPC) and GGBS. The chemical compositions of OPC and GGBS are shown in Table 2. The experiments utilized AN926SH anionic polyacrylamide (APAM) solution and calcium hydroxide [Ca(OH)_2_] as flocculants. APAM is a small white particle with a mass fraction typically ranging from 16 to 18 million. APAM particles are mixed with water at a mass ratio of 1:500 and stirred at 90 r/min until a uniform solution is formed. In addition, Ca(OH)_2_ is a fine calcium hydroxide powder.

### 2.2. Testing Procedure

One-dimensional consolidation tests of mud treated by the FSHCM are conducted in this study. The testing procedures are listed as follows:

(1) Sample mixing

The original mud and water are first mixed evenly by an electric ash mixer to obtain the required water content. Next, the desired amounts of cement and GGBS are added and stirred thoroughly. Then, the required amount of flocculant, i.e., APAM and Ca(OH)_2_, is added to prepare a mixture of flocculant, cementitious binder, and MS. The quantities of cement, GGBS, and flocculant are shown in Table 3.

(2) Making mud cakes

**Figure 3:**
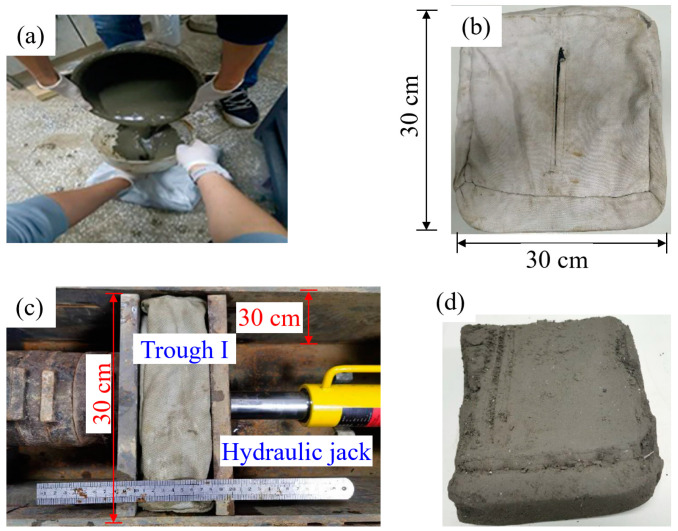
FSHCM treatment of MS: (**a**) the mixture of flocculant, cementitious binder, and MS, (**b**) the closed soil filter bag, (**c**) the pressure filtration device, (**d**) the mud cake.

As shown in Figure 3, the mixture of flocculant, cementitious binder, and MS is poured into a geo-bag. The geo-bag measured 30 cm by 30 cm in planar dimensions with a maximum thickness of 20 cm and an aperture diameter of 48 µm. Next, the geo-bag is placed into trough I of a self-designed pressure filtration device and the cover plate is fastened onto the pressure chamber by high-strength bolts. This device operates similarly to a piston, enabling the dewatering of mud slurry, as illustrated in the relevant standard [32]. Then, a hydraulic jack is used to apply pressure and facilitate dewatering. The applied pressure is 0.35 MPa, with a duration of 12 min, as shown in Figure 3c, A mud cake is obtained after completeness of pressurization. Finally, FSHCM-treated MS samples are obtained from the mud cake with cutting rings, sealed, and placed in a water bath curing box at 20 ± 3 °C for curing. The samples are cylindrical, with a diameter of 61.8 mm and a height of 20 mm. When preparing samples, avoid sampling from the edges of the mud cake. Samples with obvious defects were removed, and the specimens with similar mass and water content were selected to carry out consolidation creep tests.

(3) One-dimensional consolidation test

**Figure 4:**
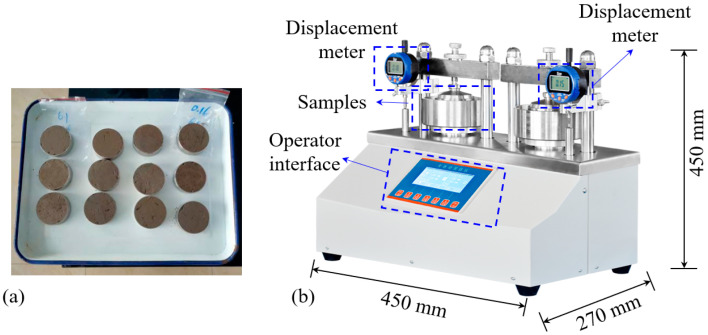
One-dimensional consolidation test: (**a**) samples, (**b**) the consolidation experimental system.

The one-dimensional high-pressure consolidation tests were carried out on FSHCM-treated MS samples cured for 7 d and 28 d. The consolidated samples and the consolidation experimental system are shown in Figure 4. The consolidation experimental system was manufactured by Shaoxing RongNa Measurement and Control Technology Co., Ltd., Shaoxing, China, with a maximum pressure of 10,000 N and a displacement measurement accuracy of 0.001 mm. The one-dimensional consolidation compression tests are conducted following the Soil Test Standard [33]. The multi-stage loading method is adopted in this test, and the vertical stress levels are 25, 50, 100, 200, 400, 800, 1600, and 2400 kPa, respectively.

### 2.3. Testing Program

The detailed test program for the one-dimensional consolidation test is listed in Table 3. Three groups of tests were conducted to explore the influences of three factors on the consolidation characteristics of FSHCM-treated mud: the dosage of the curing agent (Group A), the initial water content of the mud (Group B), and the dry weight of the MS (Group C). Each group contains four test cases, resulting in a total of twelve test cases in this experiment.

Group A: Study on the influence of curing agent content on the consolidation characteristics of FSHCM-treated MS. This group includes four test cases with fixed initial water content (200%), dry weight of the MS (2.5 kg), flocculant dosage (1.5% Ca(OH)_2_ and 0.16% APAM) but different curing agent contents, varying from 3% to 9% (mass fraction).

Group B: Study on the influence of initial water content on the consolidation characteristics of FSHCM-treated MS. The variable in this group of tests is the initial water content of the samples, which ranges from 100% to 400%.

Group C: Study on the influence of the dry weight of MS on the consolidation characteristics of FSHCM-treated MS. In the four test cases of this group, the dry weights of the MS are 3.5, 4.0, 4.5, and 5.0 kg, respectively.

## 3. Experimental Results and Discussion

### 3.1. Effects of Curing Agent Content on the Consolidation Characteristics of Treated MS

#### 3.1.1. Compression Deformation of Mud Cake

**Figure 5:**
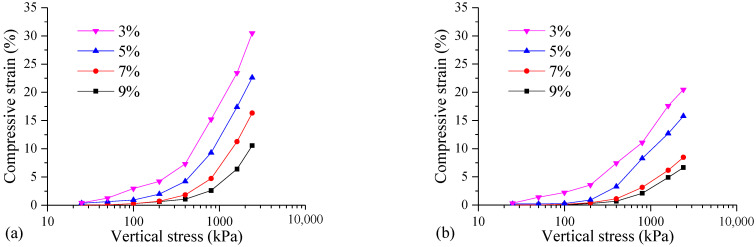
The compression deformation curves of samples with different contents of curing agent: (**a**) curing age is 7 days, (**b**) curing age is 28 days.

Based on the results of the one-dimensional consolidation tests, the compressive strain and void ratio (*e*) of the samples with different curing agent contents (3%, 5%, 7%, 9%) under various consolidation pressures were obtained. Figure 5 shows the compression deformation curves of the samples. It can be seen that for the samples with different curing agent contents, the compressive strain values of the mud cake gradually increase with the increasing vertical stress.

The curing agent contents show significant influences on the compressive strain of the FSHCM-treated MS. The compressive strain curve with a high curing agent content is consistently below that of the low curing agent content. This indicates that higher curing agent contents result in smaller compressive strain values. A higher curing agent content generates more cementitious material through the hydration reaction, leading to a stronger structure in the treated MS and greater resistance to compression deformation.

In addition, Figure 5a and Figure 5b depict the compression deformation curves of the samples cured for 7 days and 28 days, respectively. It can be seen that the compressive strain value of the samples cured for 28 days is further reduced compared to the samples cured for 7 days. Taking the sample with a curing agent content of 3% as an example, when the vertical stress levels are 25, 50, 100, 200, 400, 800, 1600, and 2400 kPa, the compressive strains of the specimens cured for 7 days are 0.42%, 1.27%, 2.96%, 4.23%, 7.32%, 15.21%, 23.45%, and 30.49%, respectively. For the samples cured for 28 days, the strains are 0.33%, 1.39%, 2.23%, 3.56%, 7.42%, 11.07%, 17.60%, and 20.47%, respectively. The results indicate that as the curing age increases, the ability of the FSHCM-treated MS to resist compressive deformation is further enhanced.

**Figure 6:**
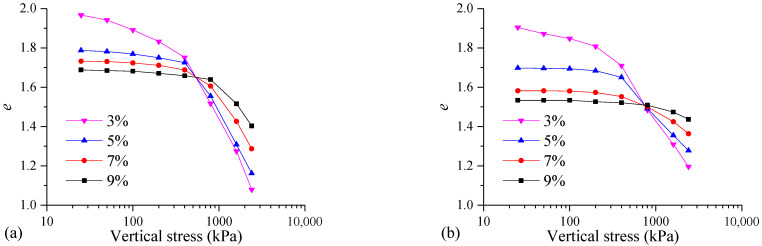
The *e*-lg*p* curves of samples with different contents of curing agent: (**a**) curing age is 7 days, (**b**) curing age is 28 days.

Figure 6 depicts the *e*-lg*p* curves of the samples cured for different times. It can be seen from Figure 6a that the higher the content of the curing agents, the smaller the initial void ratio of the sample. For the samples with curing agent contents of 3%, 5%, 7%, and 9%, the values of the initial void ratio are 1.97, 1.79, 1.73, and 1.69, respectively. With the increase in the curing agent content, the sample consumes more pore water during the curing process, and the chemical reaction produces more cementitious materials. These cementitious materials fill the pores in the soil, resulting in a smaller void ratio of the FSHCM-treated MS.

With the increase in the consolidation pressure, the void ratio of the FSHCM-treated MS with different curing agent contents gradually decreases. As the curing agent content increases from 3% to 9%, the rate of change in the void ratio of the samples decreases correspondingly. This is because a higher curing agent content means less pore water is contained in the treated MS, resulting in a more stable soil skeleton and making it more difficult to dissipate the pore water pressure under the same pressure.

In addition, the curing age significantly influences the void ratio of the mud cake. Compared with the sample cured for 7 days, the void ratio of the sample cured for 28 days is smaller. Taking the sample with a curing agent content of 3% as an example, the void ratios for the samples cured for 7 days under vertical stress levels of 25, 50, 100, 200, 400, 800, 1600, and 2400 kPa are 1.95, 1.94, 1.89, 1.83, 1.75, 1.52, 1.27, and 1.08, respectively. For the samples cured for 28 days, the void ratios under the same stress levels are 1.90, 1.87, 1.85, 1.81, 1.71, 1.48, 1.30, and 1.20, respectively. Therefore, the void ratio of the FSHCM-treated MS decreases with the increasing curing age.

#### 3.1.2. Compressibility Analysis of Mud Cake

**Figure 7:**
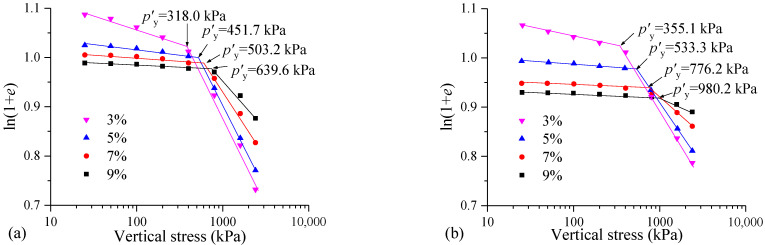
Structural yield stress *p′*_y_ of samples with different contents of curing agent: (**a**) curing age is 7 days, (**b**) curing age is 28 days.

Based on the double logarithmic method proposed by Butterfield [34], the structural yield stress (*p′*_y_) of solidified soil is determined [35,36]. Figure 7 shows the ln(1 + *e*)-lg*p* curves and *p′*_y_ values of the samples with different curing agent contents. It can be seen from Figure 7a that in the pre-yield stage, the compression curve of the sample with less curing agent content is located above. In the post-yield stage, the compression curve of the sample with a higher curing agent content is positioned above the others, indicating that the curing agent content has a significant influence on the structure of the FSHCM-treated MS. The higher the curing agent content, the smaller the influence of vertical stress on the structure of the FSHCM-treated MS. The compression curve in Figure 7b exhibits a similar trend.

Additionally, the curing age shows a significant influence on the value of *p′*_y_. When the curing agent content is 3%, 5%, 7%, and 9%, the *p′*_y_ values of the samples cured for 7 days are 318.0, 451.7, 503.2, and 639.6 kPa, respectively, while the *p′*_y_ values of the samples cured for 28 days are 355.1, 533.3, 776.2, and 980.2 kPa, respectively, showing increases of 12%, 18%, 54%, and 53%. With the increasing curing age, the ability of the FSHCM-treated MS to resist compression deformation is further enhanced.

**Figure 8:**
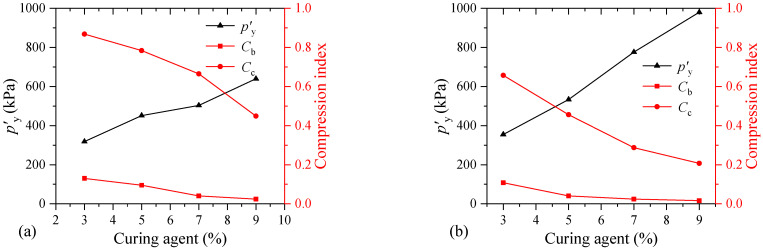
The influence of curing agent contents on the compressibility of samples: (**a**) curing age is 7 days, (**b**) curing age is 28 days.

Figure 8 depicts the changes in the structural yield stress and compression index of the samples with varying curing agent contents. *C*_c_ is the compression index of the FSHCM-treated MS. Additionally, the slope of the *e*-lg*p* curve before yielding, denoted as *C*_b_, is used to characterize the compressive properties of the FSHCM-treated MS before it reaches the structural yield stress. Considering that Figure 8 uses dual y-axis coordinates, for ease of understanding, the gray and red vertical axes represent the left and right axes corresponding to the curve readings, respectively. It can be seen from Figure 8 that the *p′*_y_ of the sample increases with the curing agent content, which is consistent with the change in the unconfined compressive strength of the solidified soil [37,38,39]. When the curing agent content increased from 3% to 9%, the *p′*_y_ values of the samples cured for 7 days and 28 days increased by 2.0 and 2.8 times, respectively. With the increase in the curing agent content, *C*_c_ and *C*_b_ gradually decrease, which is consistent with the research on the compressibility of cement-solidified soil [40]. This indicates that a higher curing agent content improves the compression resistance of the FSHCM-treated MS.

Additionally, the compression index of the sample is low before yield but increases significantly after yield. Taking the samples with a curing age of 7 days as examples, as shown in Figure 8a, for curing agent contents of 3%, 5%, 7%, and 9%, the compression indexes before yield are 0.13, 0.10, 0.04, and 0.02, respectively. After yield, the compression indexes for these samples are 0.87, 0.78, 0.66, and 0.45, respectively. The results indicate that the compressibility of the FSHCM-treated MS changes significantly at the yield point, which should be considered in practical engineering.

With the increasing curing age, the compression index of the samples decreases significantly. After being cured for 28 days, the *C*_b_ values for the samples with curing agent contents of 3%, 5%, 7%, and 9% are 0.11, 0.04, 0.02, and 0.02, respectively. Compared to the samples cured for 7 days, the *C*_b_ values decreased by 83.3%, 91.3%, 93.1%, and 90.5%, respectively. Therefore, with the increasing curing ages, the *p′*_y_ value of the sample increases while the compression index decreases. The results demonstrate that the compressibility of the FSHCM-treated MS decreases with the increasing curing age, and its ability to resist compression deformation is further enhanced.

### 3.2. Effects of Initial Water Content of Mud on the Consolidation Characteristics of Treated MS

#### 3.2.1. Compression Deformation of Mud Cake

**Figure 9:**
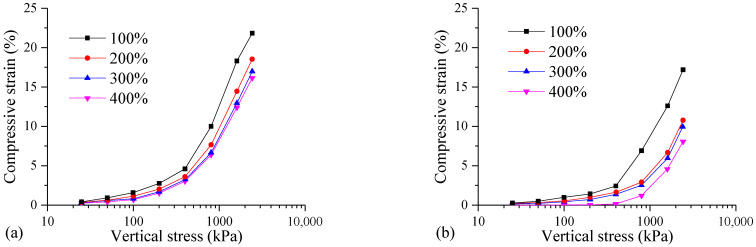
The compression deformation curves of samples with different initial water contents: (**a**) curing age is 7 days, (**b**) curing age is 28 days.

One-dimensional consolidation tests were conducted on samples with different initial water contents (100%, 200%, 300%, 400%). The compressive strain of the samples under various consolidation pressures is presented in Figure 9. Changes in the initial water content have an obvious influence on the compressive strain of the FSHCM-treated MS. The compressive strain curve of the sample with an initial water content of 100% is significantly higher than that of the other samples, with a maximum compressive strain value of 21.82%. This indicates that the samples with an initial water content of 100% have a poorer ability to resist compressive deformation. For the FSHCM-treated MS with a given curing age, the compressive strain gradually decreases with the increasing initial water content. In addition, as the curing age increases, the compressive strain of the FSHCM-treated MS decreases, enhancing its ability to resist compressive deformation.

**Figure 10:**
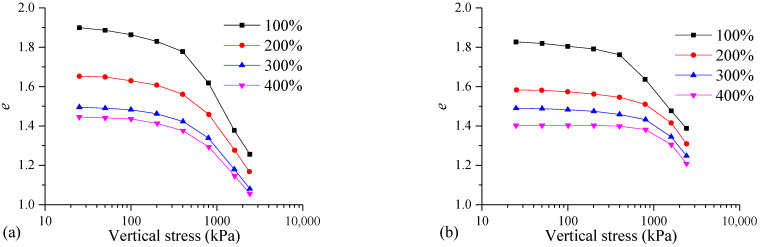
The *e*-lg*p* curves of samples with different initial water contents: (**a**) curing age is 7 days, (**b**) curing age is 28 days.

It can be seen from Figure 10 that the larger the initial water content of the MS, the smaller the initial void ratio of the FSHCM-treated MS. The higher the initial water content of the mud, the larger the flocculent pores formed in the mixed mud, making it easier to discharge pore water through high-pressure filtration, resulting in a smaller initial void ratio of the formed mud cake. With increases in the consolidation pressure, the void ratio of the samples with different initial water contents gradually decreases.

Taking the samples cured for 7 days as an example, when the vertical stress increases from 25 kPa to 2400 kPa, the changes in the void ratio for the samples with initial water contents of 100%, 200%, 300%, and 400% are 0.64, 0.48, 0.41, and 0.39, respectively. The lower the initial water content of the mud, the greater the variation in the void ratio of the samples. This is because a lower initial water content results in poorer dewatering of the mud cake, leading to more pore water in the FSHCM-treated MS. This excess pore water softens the cementitious materials, reduces the soil skeleton strength, and increases the degree of pressure change.

#### 3.2.2. Compressibility Analysis of Mud Cake

**Figure 11:**
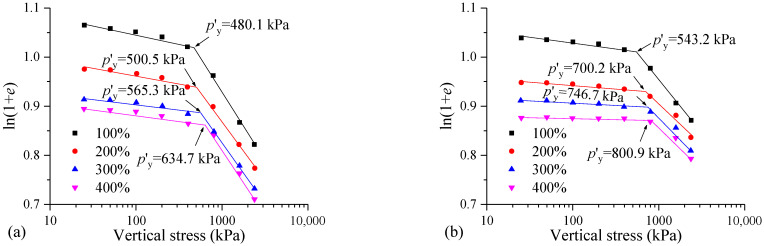
Structural yield stress *p′*_y_ of samples with different initial water contents: (**a**) curing age is 7 days, (**b**) curing age is 28 days.

Figure 11 shows the ln(1 + *e*)-lg*p* curves and *p′*_y_ values of the samples with different initial water contents. It can be seen that the higher the initial water content of the MS, the smaller the influence of the vertical stress on the FSHCM-treated MS. The curve before yield is relatively gentle, while the curve after yield is steeper. Additionally, the structural yield stress of the sample cured for 28 days is higher compared to that of the sample cured for 7 days. When the initial water contents are 100%, 200%, 300%, and 400%, the *p′*_y_ values of the samples cured for 7 days are 480.1, 500.5, 565.3, and 634.7 kPa, respectively, while the *p″*_y_ values of the samples cured for 28 days are 543.2, 700.2, 746.7, and 800.9 kPa, respectively, showing increases of 12%, 18%, 54%, and 53%.

**Figure 12:**
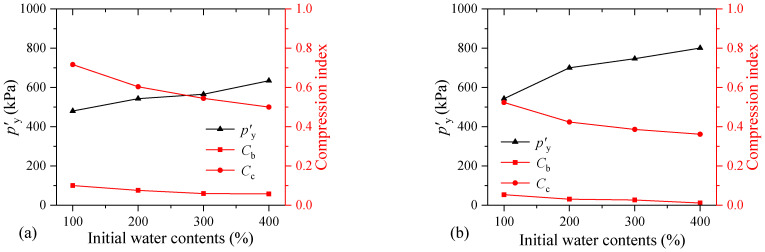
The influence of initial water contents on the compressibility of samples: (**a**) curing age is 7 days, (**b**) curing age is 28 days.

Figure 12 shows the changes in the structural yield stress and compression index of the sample with different initial water contents. It can be seen from Figure 12a that the structural yield stress of the FSHCM-treated MS increases with the initial water content. When the initial water content increases from 100% to 400%, the *p′*_y_ values of the samples cured for 7 days and 28 days increase by 1.3 and 1.5 times, respectively. Different from previous research conclusions that the compressibility of solidified soil decreases with the increasing water content, a higher initial water content in the mud results in a better effect of the FSHCM and a stronger ability of the treated MS to resist compression deformation.

Additionally, as the initial water content of the mud increases, both *C*_b_ and *C*_c_ decrease, with *C*_c_ decreasing more significantly. When the water content increases from 100% to 400%, *C*_b_ for the sample cured for 7 days decreases from 0.10 to 0.06, and *C*_c_ decreases from 0.72 to 0.50. For the sample cured for 28 days, *C*_b_ decreases from 0.05 to 0.01, and *C*_c_ decreases from 0.52 to 0.36. Furthermore, compared with the sample cured for 7 days, the structural yield stress of the sample cured for 28 days is increased, and the compression index decreases.

### 3.3. Effects of the Dry Weight of the MS on the Consolidation Characteristics of Treated MS

#### 3.3.1. Compression Deformation of Mud Cake

**Figure 13:**
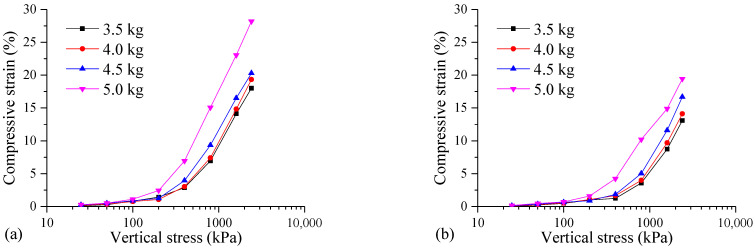
The compression deformation curves of samples with different dry weights of the MS: (**a**) curing age is 7 days, (**b**) curing age is 28 days.

One-dimensional consolidation tests were conducted on the FSHCM-treated MS samples with varying dry weights of the MS (3.5, 4.0, 4.5, and 5.0 kg). The compressive strain of the samples under different consolidation pressures is shown in Figure 13. When the vertical stress is 100 kPa, the compressive strains of the samples cured for 7 days with dry weights of 3.5, 4.0, 4.5, and 5.0 kg are 0.81%, 0.75%, 0.87%, and 1.11%, respectively, with minimal differences. As the vertical stress increases, the effects of changes in the dry weight on the compressive strain become more significant. When the vertical stress is 2400 kPa, the compressive strains of the four samples are 18.01%, 19.33%, 20.29%, and 28.20%, respectively. The greater the dry weight of the MS, the larger the compressive strain of the FSHCM-treated MS. This variation is also observed in the samples cured for 28 days.

**Figure 14:**
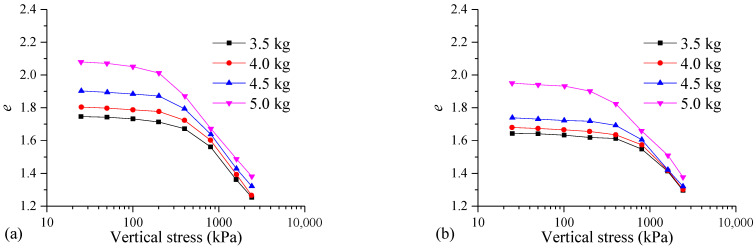
The *e*-lg*p* curves of samples with different dry weights of MS: (**a**) curing age is 7 days, (**b**) curing age is 28 days.

Figure 14 shows the *e*-lg*p* curves of the samples with different dry weights of the MS. For the samples cured for 7 days with MS dry weights of 3.5, 4.0, 4.5, and 5.0 kg, the initial void ratios are 1.75, 1.80, 1.90, and 2.08, respectively. For the samples cured for 28 days, the initial void ratios are 1.25, 1.27, 1.32, and 1.38, respectively. The results indicate that a larger dry weight of the MS corresponds to a larger initial void ratio of the FSHCM-treated MS.

The void ratio of the FSHCM-treated MS with different dry weights decreases with the increase in the consolidation pressure. A greater dry weight of the MS corresponds to a larger range of void ratios in the FSHCM-treated MS. For example, in the sample cured for 7 days, when the vertical stress increases from 25 kPa to 2400 kPa, the void ratio of the sample with a dry weight of 3.5 kg decreases from 1.75 to 1.25, a reduction of 0.50. Meanwhile, the void ratio of the sample with a dry weight of 5.0 kg decreases from 2.08 to 1.38, a reduction of 0.70. This is mainly because a greater dry weight of the MS results in a poorer dewatering effect during pressure filtration, leading to more pore water in the treated MS. This pore water softens the gelled material, making the MS easier to compress.

#### 3.3.2. Compressibility Analysis of Mud Cake

**Figure 15:**
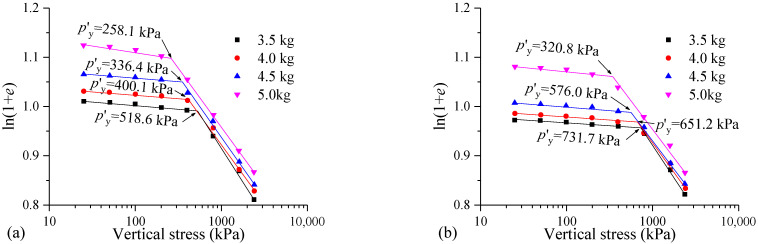
Structural yield stress *p′*_y_ of with different dry weights of the MS: (**a**) curing age is 7 days, (**b**) curing age is 28 days.

Figure 15 shows the ln(1 + *e*)-lg*p* curves and *p′*_y_ values of the samples with different dry weights of the MS. The compression curve of the FSHCM-treated MS with a greater dry weight is positioned higher in both the pre-yield and post-yield stages. In addition, the curing time has a significant impact on the *p′*_y_ value. When the dry weights of the MS are 3.5, 4.0, 4.5, and 5.0 kg, the *p′*_y_ values of the samples cured for 7 days are 518.6, 400.1, 336.4, and 258.1 kPa, respectively, while the *p′*_y_ values of the samples cured for 28 days are 731.7, 651.2, 576.0, and 320.8 kPa, respectively, showing increases of 41.1%, 62.8%, 71.2%, and 24.3%.

**Figure 16:**
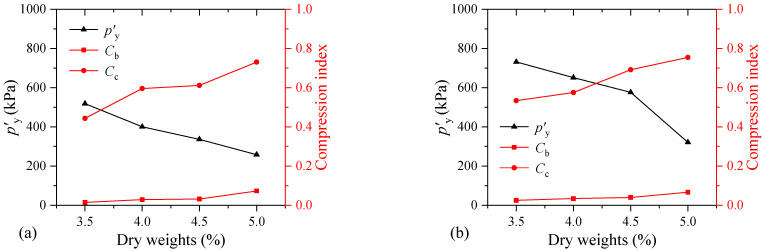
The influence of dry weights on the compressibility of samples: (**a**) curing age is 7 days, (**b**) curing age is 28 days.

Figure 16 shows the changes in the structural yield stress and compression index of the MS samples with varying dry weights. The structural yield stress of the FSHCM-treated MS decreases as the dry weight of the MS increases. When the dry weight of the MS is reduced from 5.0 kg to 3.5 kg, the *p′*_y_ values of the samples cured for 7 days and 28 days increased by 2.0 and 2.3 times, respectively. This indicates that as the dry weight of the MS increases, the ultimate external load capacity that the treated MS can resist decreases. In addition, it can be observed that *C*_b_ and *C*_c_ increase with the dry weight of the MS. Taking the sample cured for 7 days as an example, when the dry weight of the MS increases from 3.5 kg to 5.0 kg, the value of *C*_b_ increases from 0.01 to 0.07, and the value of *C*_c_ increases from 0.44 to 0.73. The results indicate that a larger dry weight of the MS results in a greater compressibility of the mud cake.

## 4. Discussion

The FSHCM integrates flocculation conditioning, chemical solidification, and physical consolidation to achieve optimal soil treatment outcomes. Curing agents can undergo hydration reactions with water, enhancing the physical and mechanical properties of mud. Adding flocculant to the soil slurry attracts soil particles and forms large flocs, resulting in a faster settlement rate compared to untreated slurry. Additionally, by applying external pressure, solid–liquid separation is achieved, effectively enhancing dehydration. The self-developed filter device is like a piston, through which the MS can be dewatered, as demonstrated in the relevant standard [32]. In addition, the consolidation tests conducted on the samples are performed according to the relevant standards [33]. Consequently, both the approach and the results reported in this manuscript can be considered repeatable and reproducible. This method is also applicable to other mud types [41]. The FSHCM enables the treatment of large volumes of MS using a plate-and-frame filter press.

The FSHCM presents significant advantages for MS treatment compared to other methods [30]. In this study, among the four groups and 16 cases, the samples from the A4 group, which were cured for 28 days, exhibited the smallest compressive strain (6.65%) and the highest *p′*_y_ value (980.2 kPa). Therefore, the optimal mix ratio of the FSHCM identified in this study is *ω*_ei_ = 200%, *M* = 2.5 kg, and *ω*_c_ = 9%, with a curing time of 28 days. The FSHCM-treated MS can be used as engineering fill material.

For the same filter press device, factors influencing the drying speed during the filter press process include the magnitude of the applied load and the loading time. For the same material, a low drying speed results in drier cakes and improved filtrate quality. At a low drying speed, pressure is gently applied to the initial slurry, enabling a slow structuring of the cake and leading to better water release [42]. Conversely, a higher drying speed produces wetter cakes and lowers the filtrate quality. In addition, the permeability of different materials varies. When subjected to the same drying rate, the dewatering performance varies across materials, resulting in differences in the water content of the samples and, consequently, variations in their consolidation behavior. In future research, it will be important to conduct compression and consolidation tests on a wider range of materials. Moreover, standardizing the magnitude and duration of the applied load will be crucial to ensure experimental accuracy.

## 5. Conclusions

In this study, a series of one-dimensional consolidation compression tests were carried out on FSHCM-treated MS, considering varying curing agent contents, initial water contents, and dry weights of the MS. The main conclusions can be summarized as follows:

(1) As the curing agent content increases, the compressive strain, void ratios, and compression indexes of the FSHCM-treated MS decrease, while the yield stress increases by at least 2.0 times when the curing agent content rises from 3% to 9%. A significant change in compressibility at the yield point should be noted in practical engineering.
(2) As the initial water content of the mud increases, the compressive strain and void ratio of the FSHCM-treated MS decrease, while the structural yield stress increases. Additionally, both C_b_ and C_c_ decrease, with a more pronounced reduction in C_c_. A higher initial water content enhances the compression resistance of the FSHCM-treated MS.
(3) The compressibility of the FSHCM-treated MS is affected by its dry weight. Increases in the dry weight of the MS result in a higher compressive strain, void ratio, and compression index for the FSHCM-treated MS. When the dry weight of the MS is reduced from 5.0 kg to 3.5 kg, the structural yield stress of the FSHCM-treated MS increases by at least 2.0 times.

## Figures and Tables

**Table 1:** Basic physical properties of tested mud.

Specific Gravity (*G*_s_)	Liquid Limit *w*_L_ (%)	Plastic Limit *w*_P_ (%)	Plasticity Index *I*_P_	Ignition Loss (%)
2.69	56.1	26.7	29.4	4.41

**Table 2:** Chemical composition of OPC and GGBS.

Material	SiO_2_	Fe_2_O_3_	Al_2_O_3_	TiO_2_	CaO	MgO	SO_3_	K_2_O	Na_2_O	Ignition Loss (%)
OPC	22.92	3.64	6.09	0.39	59.71	0.88	2.82	0.76	0.66	2.13
GGBS	30.64	0.33	15.28	0.56	36.88	5.84	0.04	0.2	0.35	0.06

**Table 3:** Program for the one-dimensional consolidation test. *ω*_ei_ represents the initial water content, *M* represents the dry weight of the MS, *ω*_c_ represents the content of curing agent, and *ω*_f_ represents the content of flocculation.

Group	Case No.	*ω*_ei_ (%)	*M* (kg)	*ω*_c_ (%)	*ω*_f_ (%)	Curing Age (Days)
A	A_1_	200	2.5	3	1.5% Ca(OH)_2_ and 0.16% APAM	7, 28
	A_2_			5		
	A_3_			7		
	A_4_			9		
B	B_1_	100	2.5	6		
	B_2_	200				
	B_3_	300				
	B_4_	400				
C	C_1_	200	3.5	6		
	C_2_		4.0			
	C_3_		4.5			
	C_4_		5.0			

## Data Availability

The original contributions presented in the study are included in the article, further inquiries can be directed to the corresponding author.
